# Advancements in bladder cancer immunotherapy: a focus on intravesical approaches

**DOI:** 10.3389/fphar.2025.1578146

**Published:** 2025-07-08

**Authors:** Xia Tang, Tianlun Yu, Hongxuan Tong, Yufan Wu

**Affiliations:** ^1^ Department of Urology, Kunshan Hospital of Traditional Chinese Medicine, Kunshan, Jiangsu, China; ^2^ Clinical Laboratory, Kunshan Rehabilitation Hospital, Kunshan, Jiangsu, China; ^3^ Institute of Basic Theory for Chinese Medicine, China Academy of Chinese Medical Sciences, Beijing, China

**Keywords:** bladder cancer, urinary bladder neoplasms, immunotherapy, immune checkpoint inhibitors, neoadjuvant immunotherapy trials, mucosal immunity, intravesical drug administration

## Abstract

Recent advances in bladder cancer immunotherapy have shown promise, particularly in addressing limitations of the current gold standard, *Bacillus* Calmette-Guérin (BCG). Novel combinations, such as sasanlimab (a PD-1 monoclonal antibody) with BCG, have improved event-free survival in high-risk non-muscle-invasive bladder cancer (NMIBC). Intravesical anti-PD-1/PD-L1 agents like pembrolizumab and nadofaragene firadenovec have demonstrated efficacy and safety in BCG-unresponsive NMIBC, leading to regulatory approval. Additionally, BCG combined with immunostimulatory protein complexes (e.g., N-803) achieved high complete response rates while preserving quality of life. For muscle-invasive bladder cancer (MIBC) patients ineligible for cisplatin, neoadjuvant immunotherapy trials are exploring anti-PD-1/PD-L1 monotherapy or combinations with anti-CTLA-4 antibodies. The Pandore trial highlights the role of mucosal immunity in predicting response to systemic immune checkpoint inhibitors. Promising results have also been observed with intravesical oncolytic immunotherapy combined with systemic anti-PD-1 therapy in cisplatin-ineligible MIBC. These advancements underscore the potential of intravesical and systemic immunotherapies to improve bladder cancer outcomes and warrant further investigation.

## Background

**FIGURE 1:**
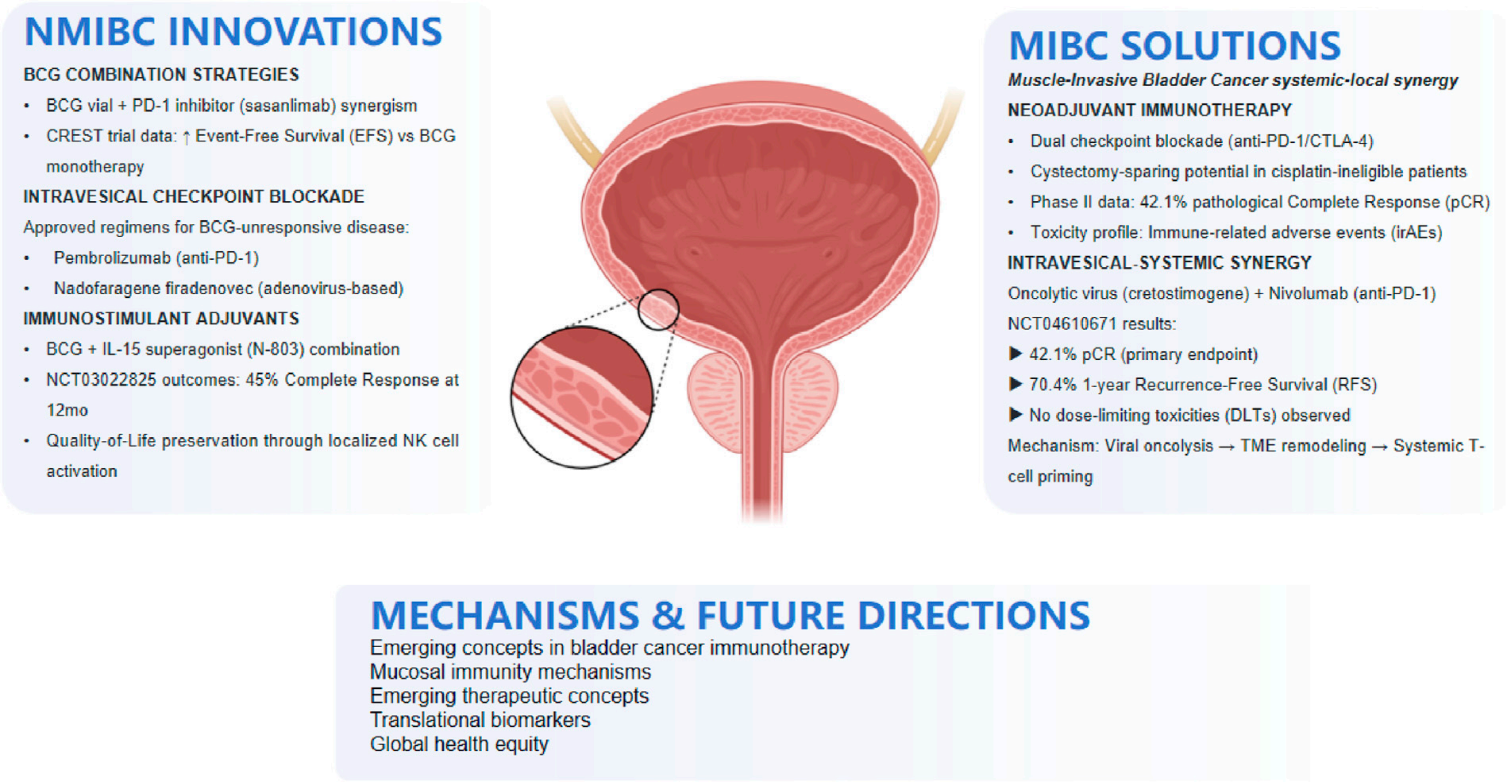
Integrated immunotherapy strategies in bladder cancer: From intravesical priming to systemic response.

Bladder cancer (BCa), the most common malignancy affecting the urinary tract, stands as one of the most prevalent cancers worldwide. Despite the relative stagnation in clinical approaches to BCa over the years, recent advancements have ushered in a new era of diagnosis and management for this disease. As a distinct disease entity, bladder cancer poses unique challenges and presents promising opportunities for immunotherapy (Kumbham et al., 2025). The bladder, a hollow organ with a direct connection to the external environment through the urethra, offers a favorable setting for the local administration of therapeutic agents directly into the bladder lumen (Lopez-Beltran et al., 2024). This intravesical route of administration offers several advantages, including high local drug concentrations, reduced systemic toxicity, and the potential to stimulate a robust mucosal immune response. Since the publication of a perspective article in 2023 that highlighted bladder cancer as a platform for drug development targeting mucosal immunity, the field has seen significant advancements (Chung et al., 2023). This article provided a comprehensive overview of the current state and future prospects of cancer immunotherapy, particularly the intravesical administration of immunostimulatory agents, laying a solid foundation for subsequent research. *Bacillus* Calmette-Guérin (BCG), the gold standard therapy for high-risk non-muscle-invasive bladder cancer (NMIBC), while effective, still results in treatment failure within 2 years in 40%–50% of patients (Naselli et al., 2024). Thus, the development of novel therapeutic strategies is of paramount importance. In the ongoing pursuit of innovative treatments, the year 2023 marked a significant milestone in clinical drug development for bladder cancer. Chung’s article revealed 32 clinical drug development projects targeting bladder cancer, with 10 of these projects already completed by early 2025, as detailed in Table 1. These completed projects have not only enhanced our understanding of bladder cancer but have also laid the groundwork for advancements in immunotherapies. Figure 1 provides an overview of integrated immunotherapy strategies in bladder cancer, highlighting the synergy between intravesical priming and systemic immune modulation, along with emerging therapeutic innovations and future directions.

**TABLE 1:** Overview of novel therapeutic approaches for bladder cancer in clinical trials.

Types	Compound	Drug administration	Disease setting	Phase	NCT number
Viral vectors	Coxsackievirus A21± MMC	Intravesical	Neoadjuvant prior to TURBT/NMIBC	II	NCT02316171 (Annels et al., 2019)
Viral vectors	Attenuated Measles Virus	Intravesical	Neoadjuvant prior to RC	I	NCT03171493
Viral vectors	Recombinant Adenovirus + Pembrolizumab	Intravesical + I.V.	Adjuvant/BCG-unresponsive HR NMIBC	II	NCT04387461
Viral vectors	Recombinant Adenovirus-IFN	Intravesical	Adjuvant/BCG-unresponsive HR NMIBC	III	NCT02773849 (Boorjian et al., 2021)
Synthetic chemo-compounds	Recombinant DNA Diphteria Toxin + BCG	Intravesical	Adjuvant/NMIBC	I/II	NCT01878188
Synthetic chemo-compounds	Recombinant BCG	Intravesical	Adjuvant/BCG-unresponsive NMIBC	I/II	NCT02371447 (Rentsch et al., 2022)
Synthetic chemo-compounds	Recombinant Bacterial Minicells	Intravesical	Adjuvant/NMIBC	I	NCT03854721
Immunomodulators	Imiquimod	Intravesical	Adjuvant/Carcinoma *In Situ* (CIS)	II	NCT01731652 (Donin et al., 2017)
Immunomodulators	Recombinant Melanoma-associated antigen 3 (MAGE-A3) protein + BCG	I.V. + Intravesical	Adjuvant/NMIBC	I	NCT01498172
Immunomodulators	Rapamycin + BCG	P.O. + Intravesical	Adjuvant/BCG-naïve HR NMIBC	I	NCT02753309 (Ji et al., 2021)

This table summarizes the clinical trials employing various compounds, drug administration routes, and disease settings for different types and stages of bladder cancer. Each row represents a specific clinical trial, including the type of therapy used (such as viral vectors, synthetic chemo-compounds, immunomodulators, etc.), the name of the compound or drug, the route of administration, the disease setting (such as neoadjuvant therapy, adjuvant therapy, etc.), the phase of the clinical trial, and the NCT (National Clinical Trial) number. These data provide an overview of novel therapeutic methods currently under investigation for bladder cancer patients and treatment providers. All clinical trials are cited via NCT, identifiers. Published studies are referenced in the text; unpublished data may be accessed through ClinicalTrials.gov or PubMed using the provided identifiers. BCG (*Bacillus* Calmette-Guérin), TURBT (Transurethral Resection of Bladder Tumor), RC (Radical Cystectomy), NMIBC (Non-Muscle Invasive Bladder Cancer), HR (High-Risk), CIS (Carcinoma *In Situ*), I.V. (Intravenous), and P.O. (Per Os/Oral).

## Overview of recent advancements

As of early 2025, the progress in bladder cancer immunotherapy is encouraging. Recently, Pfizer announced that its PD-1-targeted monoclonal antibody, sasanlimab, met the primary endpoint in the pivotal Phase 3 CREST trial1. When combined with BCG as induction therapy (with or without maintenance therapy), sasanlimab significantly improved event-free survival (EFS) in BCG-naïve, high-risk NMIBC patients. Data from studies on intravesical administration of anti-PD-1/PD-L1 monoclonal antibodies have demonstrated their efficacy and safety in BCG-unresponsive patients, further confirming their potential as neoadjuvant treatment options (Steinberg et al., 2024). The first-in-human trial validated the feasibility and safety of intravesical pembrolizumab injection for BCG-unresponsive cancer patients (Meghani et al., 2022). Notably, while systemic PD-1 blockade has shown limited efficacy in such patients, intravesical administration elicited a robust immune response at the bladder mucosal surface without significant systemic toxicity. To date, two agents (pembrolizumab and nadofaragene firadenovec) have been approved (Guerrero-Ramos et al., 2024). This strategy not only triggers local anti-tumor immunity but also stimulates a systemic adaptive anti-tumor immune response in preclinical and early clinical studies, highlighting its investigational potential as a neoadjuvant therapy.

## Combination therapy with BCG and immunostimulatory protein complexes

In clinical trials, the combination therapy of BCG with immunostimulatory protein complexes, such as N-803, has shown significant efficacy, providing new treatment options for NMIBC patients unresponsive to BCG therapy (Chamie et al., 2024a). The strategy of combining BCG with IL-15-based immunostimulatory protein complexes (like N-803) has made breakthrough progress in addressing the challenge of treating BCG-unresponsive patients. In the multicenter trial NCT03022825 (Chamie et al., 2024b), the combination therapy achieved a complete response rate of up to 45% at 12 months. This synergistic therapeutic effect may be attributed to N-803’s ability to activate natural killer (NK) cells, effectively combating MHC Class I-deficient cancer cells, thereby compensating for BCG’s deficiencies in inducing an immune response and improving patient outcomes and quality of life. Furthermore, the trial focused on patient-reported outcomes (PROs), showing that physical function (PF) and global health (GH) scores remained relatively stable during treatment, further confirming the advantages of this combination therapy in maintaining patient quality of life.

### Neoadjuvant immunotherapy for muscle-invasive bladder cancer (MIBC)

Trials on neoadjuvant immunotherapy for muscle-invasive bladder cancer (MIBC) patients, particularly those ineligible for cisplatin, are deepening, aiming to enhance treatment efficacy and reduce toxicity. Although radical cystectomy combined with neoadjuvant cisplatin chemotherapy is the standard treatment, many patients are unsuitable for cisplatin due to high toxicity or underlying comorbidities. Therefore, emerging neoadjuvant trials are exploring the efficacy of anti-PD-1/PD-L1 monotherapy or combination therapy with anti-CTLA-4 monoclonal antibodies before radical cystectomy in cisplatin-ineligible patients.

Recent trials underscore the evolving role of neoadjuvant immunotherapy in cisplatin-ineligible MIBC, though toxicity management remains a priority. Grivas et al. (2021) evaluated neoadjuvant nivolumab (anti-PD-1) monotherapy or combined with lirilumab (anti-KIR) in cisplatin-ineligible patients, reporting dose-limiting toxicities (DLTs) that highlight the need for optimized regimens. Gazzoni et al. (2025) demonstrated that dual PD-1/PD-L1 and CTLA-4 inhibition increased pathological complete response (pCR) rates (42.1% vs. 29.2% for monotherapy), though grade ≥3 adverse events (e.g., colitis, hepatotoxicity) occurred in 35% of patients, limiting broader adoption. Anti-PD-1/PD-L1 monotherapy has shown moderate efficacy in cisplatin-ineligible cohorts. Necchi et al. (2018) reported a 34% pCR rate with pembrolizumab in high-risk MIBC, while Guercio et al. (2022) observed that nivolumab + ipilimumab combinations elevated pCR to 52.1% but incurred a 31.8% rate of severe toxicities, necessitating safer alternatives.

Innovative strategies integrating intravesical and systemic therapies aim to balance efficacy and safety. Li R. et al. (2022) combined CG0070 (GM-CSF-expressing oncolytic adenovirus) with systemic nivolumab, achieving a 42.1% pCR in cisplatin-ineligible MIBC. CG0070 acted as an “*in situ* vaccine” via tumor lysis and immune activation, synergizing with nivolumab without exacerbating systemic toxicity. Similarly, Cathomas et al. (2024) explored sequential intravesical BCG followed by chemo-immunotherapy, where BCG-induced PD-L1 upregulation enhanced subsequent PD-1/PD-L1 blockade efficacy, reducing chemotherapy reliance. Psutka et al. (2023) reported that TAR-200 (intravesical drug-eluting device) plus cetrelimab (anti-PD-1) achieved localized immune activation with minimal systemic exposure, offering a toxicity-reduction pathway. These data collectively support the rationale for localized immune priming (e.g., oncolytic viruses, BCG) to amplify systemic checkpoint inhibitor responses, thereby improving therapeutic indices in cisplatin-ineligible MIBC.

While these trials report generally favorable efficacy, they also highlight substantial toxicity issues, emphasizing the need to develop less toxic and more effective treatment strategies. Notably, the combination of intravesical delivery of immunotherapy with systemic immune checkpoint inhibition offers a novel and exciting treatment approach for MIBC patients.

## Intravesical oncolytic immunotherapy and systemic immune checkpoint inhibition

The Pandore clinical trial indicates a close correlation between mucosal immunity and responsiveness to systemic immune checkpoint inhibitors. Exciting preliminary results have emerged from ongoing trials, such as NCT04610671 (Li et al., 2025), which evaluate the efficacy and safety of intravesical oncolytic viruses or chemotherapy combined with anti-PD-1 monoclonal antibodies in cisplatin-ineligible muscle-invasive bladder cancer (MIBC) patients. This trial assessed the efficacy and safety of intravesical injection of an oncolytic virus (cretostimogene grenadenorepvec, an oncolytic adenovirus type 5 encoding granulocyte-macrophage colony-stimulating factor) combined with systemic use of the anti-PD-1 monoclonal antibody nivolumab. Among the 21 enrolled and treated patients, no dose-limiting toxicity was observed. The combination therapy achieved a pathological complete response rate of 42.1% and a 1-year recurrence-free survival rate of 70.4%. Key efficacy and safety outcomes from the NCT04610671 trial are summarized in Table 2.

**TABLE 2:** Efficacy and safety outcomes of intravesical cretostimogene grenadenorepvec + systemic nivolumab in cisplatin-ineligible MIBC (NCT04610671).

Outcome measure	Result	Notes
Study Population	n = 21 (enrolled/treated)	Cisplatin-ineligible MIBC patients
Pathological Complete Response (pCR)	8/19 (42.1%)	Primary efficacy endpoint; evaluated in 19 efficacy-assessable patients
1-Year Recurrence-Free Survival (RFS)	0.704	Kaplan-Meier estimate; median follow-up: 15.2 months
Dose-Limiting Toxicity (DLT)	0/21 (0%)	Primary safety endpoint met
Tertiary Lymphoid Structures (TLS)	Increased in responders	Correlated with pCR (p < 0.01); indicates coordinated humoral-cellular immunity

Although intravesical oncolytic immunotherapy broadly induced T-cell infiltration, the formation, expansion, and maturation of tertiary lymphoid structures were particularly associated with complete responses, supporting the importance of a coordinated humoral and cellular immune response. Collectively, these results highlight the potential of this combination therapy to enhance treatment efficacy in cisplatin-intolerant MIBC patients and warrant further investigation as a neoadjuvant treatment option.

## Future prospects

The trajectory of bladder cancer immunotherapy is poised for transformative advancement, driven by innovative intravesical-systemic synergies, precision medicine strategies, and global health equity initiatives. Emerging “prime-and-boost” paradigms, exemplified by intravesical oncolytic viruses combined with systemic anti-PD-1 agents, demonstrate enhanced T-cell activation through localized immunogenic cell death and systemic checkpoint blockade (Li X. et al., 2022). Such approaches may be further optimized by coupling intravesical adenoviruses encoding cytokines with bispecific antibodies targeting immune checkpoints, offering potential to overcome immunosuppressive barriers like DC-SIGN + macrophages in the tumor microenvironment (TME).

Precision immunotherapy will be refined by liquid biopsy technologies, such as urinary tumor DNA (utDNA) profiling, which enables real-time monitoring of therapeutic response and early relapse detection (Zhang et al., 2023). Concurrently, biomarkers like CD39 expression and immunoproteasome signatures hold promise for patient stratification, as validated in retrospective analyses of neoadjuvant trials. Prospective integration of these tools into trials could minimize toxicities by excluding non-responders (Cathomas et al., 2024).

Overcoming intrinsic resistance mechanisms remains critical, with emerging strategies targeting TME suppressors: anti-CCR4 antibodies for Treg depletion, CSF-1R inhibitors to counter MDSC activity, and IDO1/adenosine pathway modulators for metabolic reprogramming. For cisplatin-ineligible MIBC, bladder-sparing regimens—such as TAR-200 (intravesical drug-eluting device) plus cetrelimab—may redefine standards of care by balancing local control and quality of life (Williams et al., 2021).

Finally, addressing global disparities in BCG access necessitates scalable innovations, including recombinant BCG strains (e.g., VPM1002BC) and live-attenuated *Listeria* vectors expressing tumor antigens (Rentsch et al., 2022). Phase II data affirm comparable efficacy to conventional BCG, with logistical advantages for resource-limited settings. Collectively, these advancements promise to reshape bladder cancer care, fostering durable cures while prioritizing equity and patient-centered outcomes.

## Conclusion

The field of bladder cancer immunotherapy is experiencing a transformative period, marked by significant advancements in intravesical approaches. The integration of novel agents like sasanlimab with BCG has demonstrated improved outcomes in high-risk NMIBC, while intravesical anti-PD-1/PD-L1 therapies have shown promise in BCG-unresponsive patients, leading to regulatory approvals. The combination of BCG with immunostimulatory protein complexes, such as N-803, has achieved high complete response rates while preserving patient quality of life. For MIBC patients ineligible for cisplatin, neoadjuvant immunotherapy trials are exploring anti-PD-1/PD-L1 monotherapy or combinations with anti-CTLA-4 antibodies, with intriguing results observed in trials combining intravesical oncolytic immunotherapy with systemic anti-PD-1 therapy. These developments underscore the potential of intravesical and systemic immunotherapies to revolutionize bladder cancer treatment paradigms. Future research should focus on refining these therapies to enhance efficacy, reduce toxicity, and expand treatment options for a broader patient population, ultimately improving outcomes and quality of life for those with bladder cancer. The ongoing evolution of immunotherapy in bladder cancer holds great promise and warrants continued investigation to fully realize its potential.
